# Clinical characteristics and risks of Chinàs 2019 novel coronavirus patients with AKI: a systematic review and meta-analysis

**DOI:** 10.1080/0886022X.2020.1812401

**Published:** 2020-09-02

**Authors:** Zhiqin Zhang, Lian Zhang, Dongqing Zha, Chun Hu, Xiaoyan Wu

**Affiliations:** Nephrology Department, Zhongnan Hospital of Wuhan University, Wuhan, China

Since December 2019, there has been an increasing number of unexplained cases of pneumonia in Wuhan, a city with 11 million people in China's Hubei province, which quickly spread to other cities and also abroad. These cases with laboratory confirmed a viral infection have been detected in the world and the World Health Organization (WHO) called this virus as COVID-19 .Given the rapid spread and high mortality rate of COVID-19 with acute kidney injury (AKI), it is absolutely necessary to evaluate the clinical characteristics and possible risk factors affecting the progression of disease in COVID-19 patients with AKI.

The clinical data of COVID-19 patients from 1 December 2019 to 30 June 2020 were retrieved from databases, including PubMed, Embase, Web of Science, WanFang Data, CNKI, and medrxiv. We statistically analyzed the clinical characteristics, symptoms and examination`s results of COVID-19 patients and explained the clinical features of a meta-analysis. The available data of 789 patients in four publications were included in our meta-analysis [1–4]. The common clinical symptoms of COVID-19 patients with AKI were fever, cough, myalgia or fatigue which is the same as COVID-19 patients with non-AKI (NAKI). But what`s more common than general COVID-19s than the patients with AKI was hypoxia. The results of laboratory results showed that the COVID-19 patients with AKI had higher procalcitonine besides increased lymphocytopenia, C-reactive protein (CRP), lactate dehydrogenase (LDH) and leukocytopenia that represented more inflammation. Meanwhile our meta-analysis found the male with underlying disease like diabetes, chronic obstructive pulmonary disease (COPD), chronic kidney disease (CKD), cerebrovascular or cardiovascular disease were more likely to get AKI. As we can see those patients were also more likely to have lower discharge rate and higher fatality rate. The results of the meta-analysis showed that: male (OR:3.43 [1.38, 8.49], *p* < 0.05), diabetes (OR:2.63 [1.05, 6.58], *p* < 0.05), COPD (OR: 2.98 [1.27, 6.98], *p* < 0.05), CKD (OR:3.26 [1.03, 10.32], *p* < 0.05), cardiovascular disease (OR: 2.26 [1.21, 4.21], *p* < 0.05), and cerebrovascular disease (OR:2.95 [1.38, 6.32], *p* < 0.05) were independent risk factors associated with COVID-19 patients with AKI. The meta-analysis revealed no correlation between increased risk of COVID-19 and hypertension, cancer, or chronic liver disease. The discharge rate of COVID-19 patients with AKI was 0.2 [0.11,0.36], *p* < 0.001 and the fatality rate was 8.78 [5.04,15.32], *p* < 0.001.

Four studies included in quantitative synthesis (Figure 1). Data extraction and the evaluation of literature quality were extracted independent (Table 1). The MINORS (Table 2) was used to evaluate bias risk. The Excel was used to analyze the clinical symptoms and examination results. Meta-analysis was performed using Revman5.3.5. When heterogeneity I^2^ < 50%, a fixed-effects model was chosen, while when I^2^> 50%, a random-effects model was selected. we consider *p* < 0.05 as statistical significance (2-sided).

**Figure 1.**
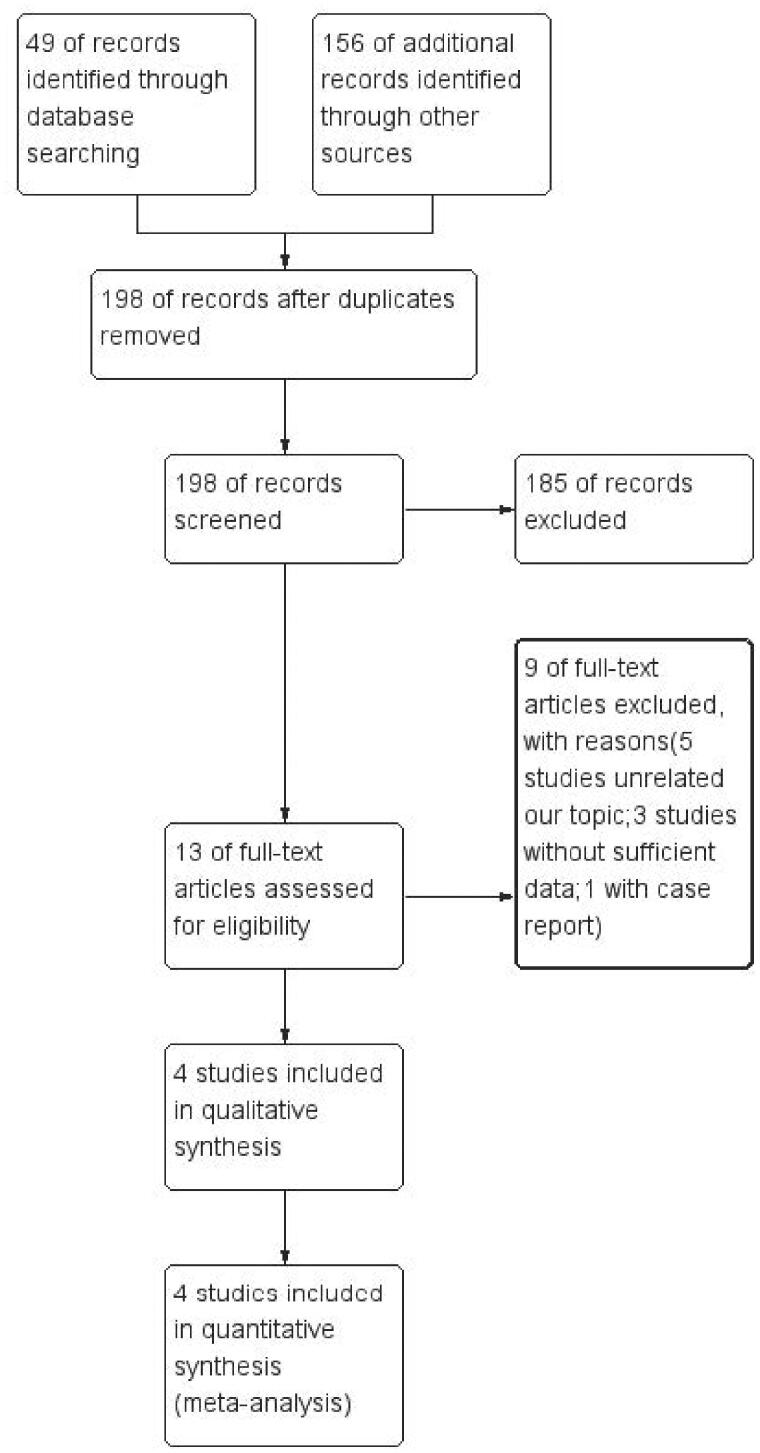
A flow diagram of the inclusion criteria of studies eligible for meta-analysis.

**Table 1.** Demographics of the included studies.

Study	Year	Country	Number of patients	hospital	AKI	NAKI
Xu S.2020 [1]	2020	china	62	Zhongnan Hospital Affiliated to Wuhan University	18	44
S.L 2020 [2]	2020	china	355	Union Hospital of Huazhong University of Science and Technology	56	299
Bo D. 2020 [3]	2020	china	85	General Hospital of Central Theater Command	23	62
Guanhua X. 2020 [4]	2020	china	287	Hankou Hospital	55	232

**Table 2.** Bias risk assessment.

Study	①	②	③	④	⑤	⑥	⑦	⑧	score
Xu S.2020	2	2	2	2	2	1	1	0	12
S.L 2020	2	2	2	2	2	1	2	0	13
Bo D. 2020	2	2	2	2	1	1	1	0	11
Guanhua X. 2020	2	2	2	2	2	2	2	0	14

① A clearly stated aim; ② Inclusion of consecutive patients; ③ Prospective collection of data; ④ Endpoints appropriate to the aim of the study; ⑤ Unbiased assessment of the study endpoint; ⑥ Follow-up period appropriate to the aim of the study; ⑦ Loss to follow up less than 5%; ⑧ Prospective calculation of the study size. The items are scored 0 (not reported), 1 (reported but inadequate) or 2 (reported and adequate). The global ideal score being 16 for non-comparative studies.

## Male and diabetes

**Figure 2.**
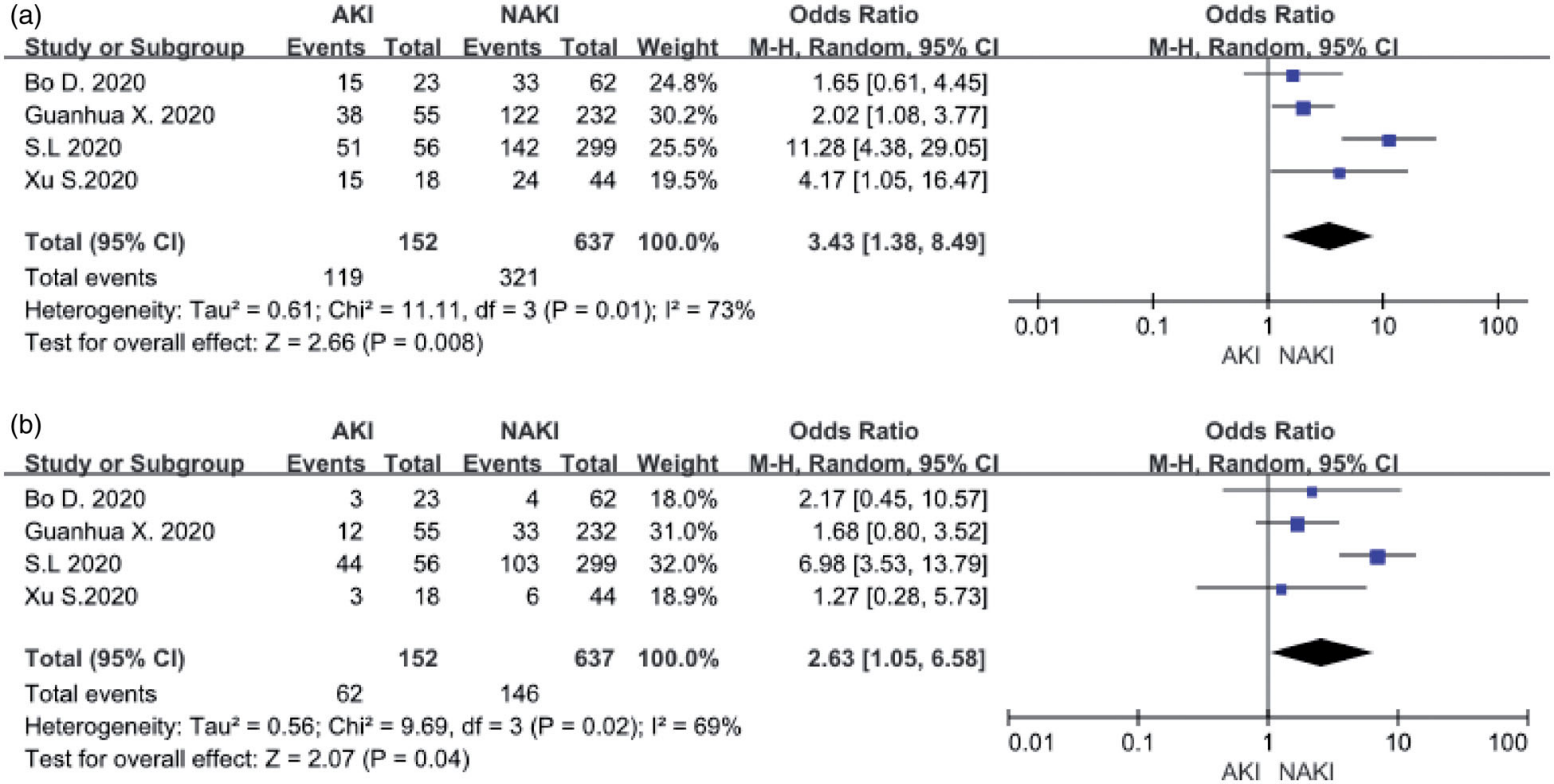
(A) Male distribution. (B) Diabetes distribution.

Four studies, including 152 COVID-19 patients with AKI and 637 COVID-19 patients with non-AKI (NAKI), provided the data in terms of male and diabetes. The heterogeneity test showed high heterogeneity among these studies, and random-effects model was used for the meta-analysis. The results find that COVID-19 patients with male (OR:3.43, 95%CI [1.38, 8.49], *p* < 0.05) (Figure 2(A)) and diabetes (OR:2.63, 95%CI [1.05, 6.58], *p* < 0.05) (Figure 2(B)) had a higher risk of AKI.

## COPD, CKD, cerebrovascular disease and cardiovascular disease

**Figure 3.**
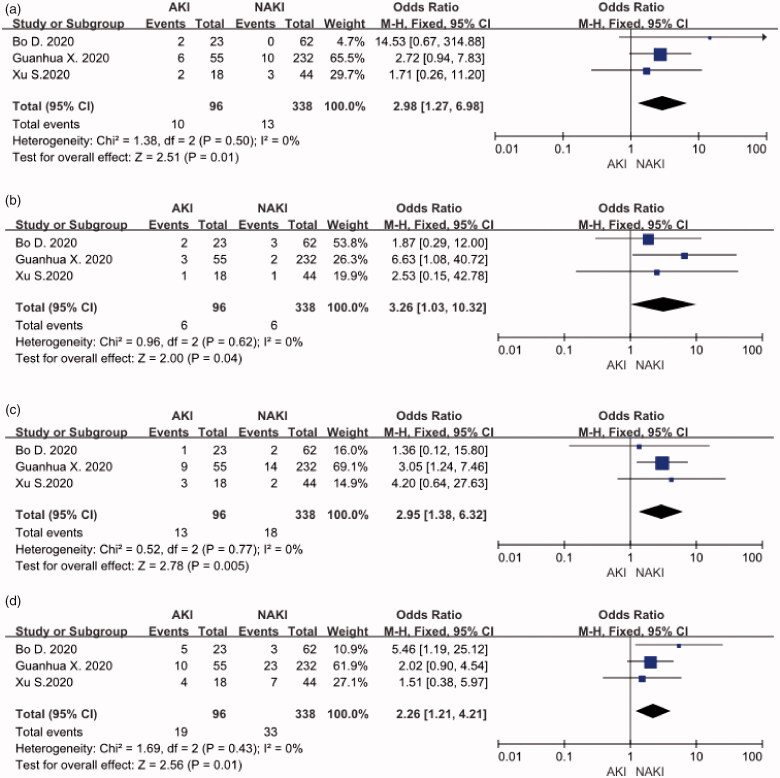
(A) COPD Distribution. (B) CKD Distribution. (C) Cerebrovascular disease distribution. (D) Cardiovascular disease distribution.

Three included studies reported relationships between COPD, CKD, cardiovascular disease, and COVID-19 patients with AKI. No significant heterogeneity was found (I2 = 0) among these trials, so a fixed effect pattern was chosen. The results showed that these risk factors for patients with COVID-19 and AKI. COPD (OR: 2.98, 95%CI [1.27, 6.98], *p* < 0.05), CKD (OR:3.26, 95%CI [1.03, 10.32], *p* < 0.05), cardiovascular disease (OR: 2.26, 95%CI [1.21, 4.21], *p* < 0.05), and cerebrovascular disease (OR:2.95, 95%CI [1.38, 6.32], *p* < 0.05) are seen in these meta-analysis respectively. (Figure 3(A–D)).

## Hypertention, cancer and chronic liver disease

**Figure 4.**
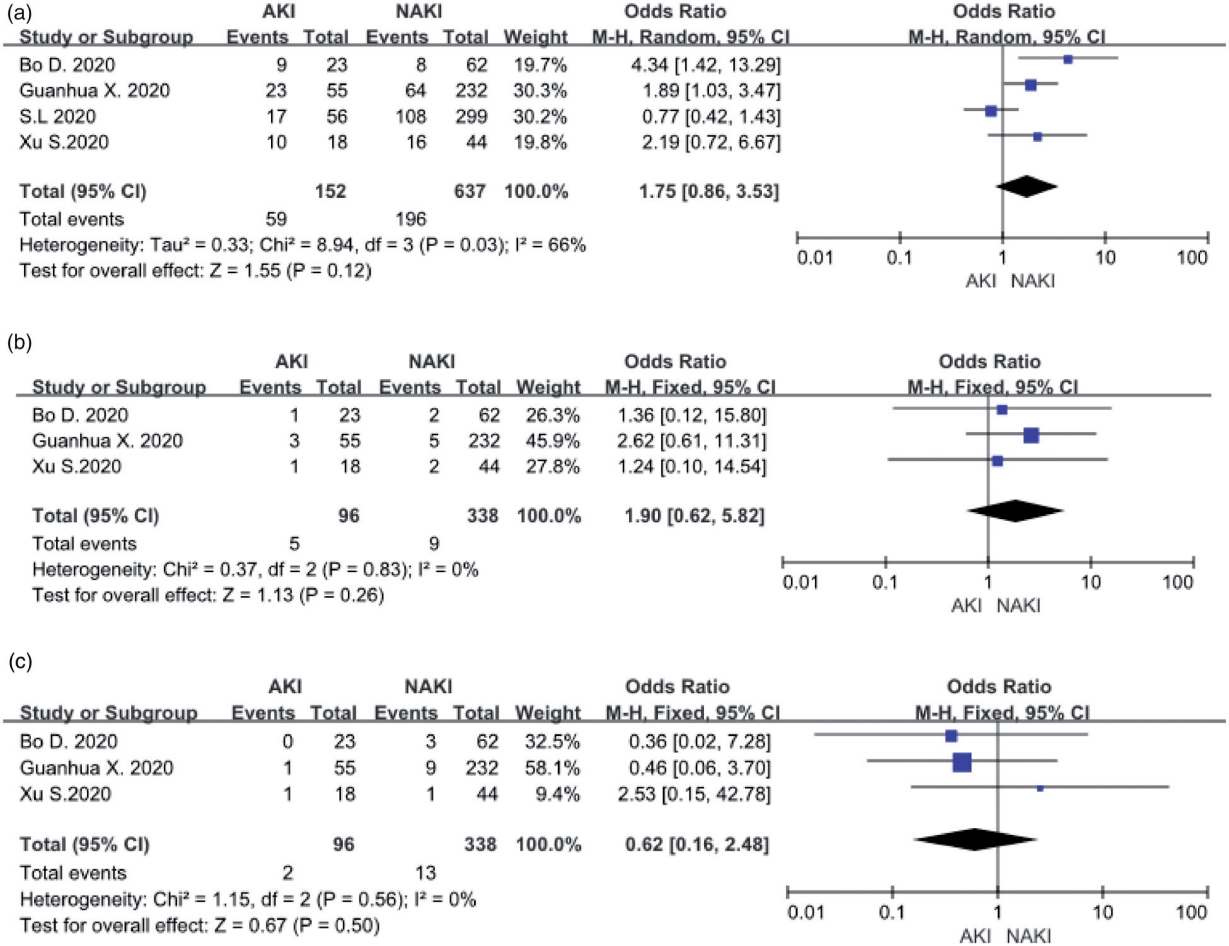
(A) Hypertension distribution. (B) Cancer distribution. (C) Chronic liver disease distribution.

Four studies comprising 152 COVID-19 patients with AKI and 637 COVID-19 patients with NAKI evaluated the role of hypertension in patients with COVID-19 and AKI. The meta-analysis showed that patients with the hypertension did not increase the risk of disease progression (OR:1.75, 95%CI[0.86,3.53], *p* = 0.12) (Figure 4(A)).The relative risk assessments associated with cancer and chronic liver disease are presented in Figure 4(B,C). The meta-analysis suggested that there was also no correlation between tumor (OR:1.9, 95%CI[0.62,5.82], *p* = 0.26) or hepatobiliary disease (OR:0.62, 95%CI[0.16,2.48], *p* = 0.5) and COVID-19 patients with AKI.

## Discharge rate and fatality rate

**Figure 5.**
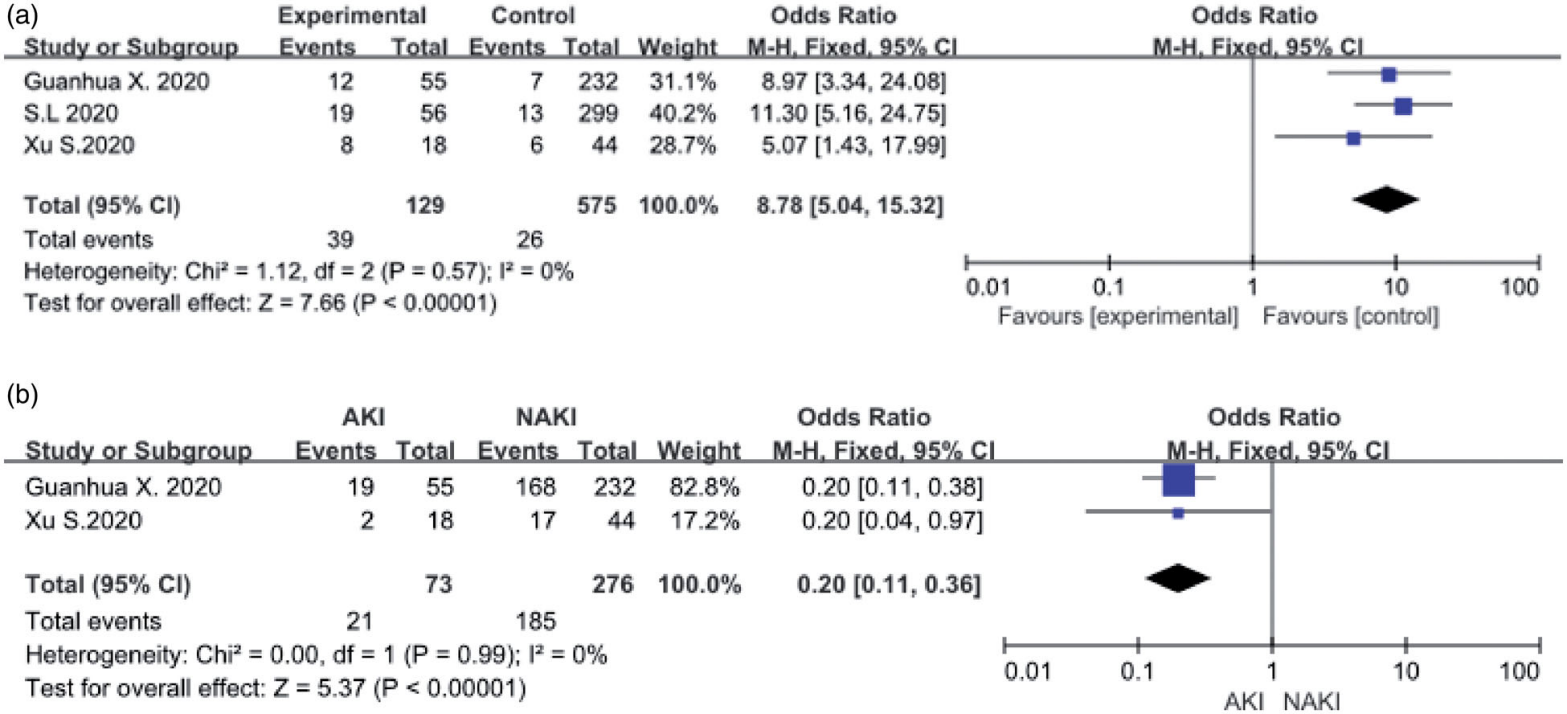
(A) Fatality rate. (B) Discharge Rate.

The three studies showed results of the meta-analysis which revealed the fatality rate of the COVID-19 patients with AKI (OR:8.78, 95%CI [5.04,15.32], *p* < 0.001). (Figure 5(A)) A fixed effect pattern was chosen because the low heterogeneity I^2^ = 0. The two studies showed results of the fixed effects model meta-analysis that revealed the discharge rate of the COVID-19 patients with AKI (OR: 0.2,95%CI [0.11,0.36], *p* < 0.001) (Figure 5(B)) compared with pure COVID-19 patients, COVID-19 and AKI patients do not have much different on the symptoms [5–7]. They all have a fever, cough, dizziness, etc. but according to the data in literature, we do focus hypoxia that is easier to emerge in AKI. It means that we need to give oxygen treatment early. In laboratory tests, we also notice PCT, in which many items represent inflammation, could be a effective diagnosis of COVID-19 and AKI (Tables 3,4).

**Table 3.** Clinic syndrome and therapy.

Study		Fever	Cough	Expectoration	Sore throat	Nasal Congestion	Myalgia or Fatigue	Headache or Dizziness	Diarrhea	Nausea and vomiting
Xu S.2020	AKI	12 (66.7)	3 (16.7)	7 (38.9)	0 (0)	–	2 (11.1)	2 (11.1)	1 (5.6)	0 (0)
	NAKI	36 (81.8)	11 (25.0)	11 (27.3)	2 (4.5)	–	13 (29.5)	3 (6.8)	1 (2.3)	3 (6.8)
	P	0.201	0.484	0.282	0.366		0.081	0.58	0.515	0.083
Guanhua X. 2020	AKI	40 (73)	41 (75)	–	–	–	25 (45)	–	–	–
	NAKI	183 (79)	168 (72)	–	–	–	112 (48)	–	–	–
	p	0.32	0.75	–	–	–	0.71	–	–	–

Study		Dyspnea	Hypoxia*	Tachypnea*	Nasal catheter/high flow oxygen therapy	Noninvasive mask*	Endotracheal intubation/ tracheotomy*	ECMO	Invasive ventilation*
Xu S.2020	AKI	–	–	–	3 (16.7)	2 (11.1)	12 (66.7)	2 (11.1)	14 (77.8)
	NAKI	–	–	–	10 (22.7)	17 (38.6)	8 (18.2)	8 (18.2)	16 (36.4)
	P	–	–	–	0.602	0.013	0.001	0.467	0.003
Guanhua X. 2020	AKI	29 (53)	27 (49)	10 (18)	–	–	–	–	–
	NAKI	110 (47)	72 (31)	11 (5)	–	–	–	–	–
	p	0.48	0.01	0.01	–	–	–	–	–

*p < 0.05.

**Table 4.** Clinic examination and result.

Study		WBC* (×109/L)	Hb (g/L)	LYN (×109/L)*	D-dimer* (mg/L)	ALT (U/L)	AST (U/L)*	LDH (U/L)*	CRP (mg/L)*	PCT (ng/mL)*
Xu S.2020	AKI	10.7 ± 5.7	123.3 ± 23.2	0.7 ± 0.4	5223.7 ± 588.2	52.6 ± 12.5	72.2 ± 18.8	572.4 ± 196.9	91.1 ± 21.6	1.2 ± 0.7
	NAKI	9.0 ± 5.3	117.9 ± 22.0	0.5 ± 0.3	4260.5 ± 516.8	43.2 ± 20.6	58.7 ± 19.0	461.4 ± 187.4	89.9 ± 12.1	8.5 ± 2.9
	P	–	–	–	–	–	–	–	–	<0.05
Guanhua X. 2020	AKI	5.8 (4.3-9.5)	125 ± 20	4.5 (3-8.5)	0.9 (0.2-6.6)	27 (18-43)	35 (23-50)	344 (234-469)	34.6 (10.6-71.7)	0.15 (0.09-0.41)
	NAKI	4.9 (3.6-6.7)	129 ± 16.3	3.4 (2.2-5.2)	0.5 (0.2-1.6)	24 (16-37)	26 (19-40)	238 (183-333)	19.5 (4-37)	0.07 (0.04-0.13)
	p	<0.01	0.11	<0.01	<0.01	0.18	<0.01	<0.01	0.02	<0.01

*p < 0.05; WBC: White blood cell; Hb: Hemoglobin; LYN: Nymphocyte; ALT: Alanine aminotransferase; AST: Aspartate aminotransferase; LDH: Lactate dehydrogenase; CRP: C-reactive protein; PCT: Procalcitonin.

We made a conclusion that we need pay attention to the degree of hypoxia, inflammatory biomarkers especially PCT of COVID-19 and AKI. And we also should focus on men, diabetes mellitus, COPD, chronic kidney disease (CKD), cardiovascular disease, cerebral blood coronary disease risk factors so that accurate treatment can be made up according to the different condition [8–10]. The meta has some defects: the articles gathered in this literature are all from China, and the size of the sample is not big enough. So the result needs to be further in-depth study.
